# Successful Reimplantation of Spinal Cord Stimulator One Year after Device Removal Due to Infection

**DOI:** 10.1055/s-0040-1722179

**Published:** 2021-02-01

**Authors:** Ruben H. Schwartz, Warren Southerland, Ivan Urits, Alan D. Kaye, Omar Viswanath, Cyrus Yazdi

**Affiliations:** 1 Department of Anesthesiology, Mount Sinai Medical Center, Miami Beach, Florida; 2 Department of Anesthesia, Critical Care, and Pain Medicine, Beth Israel Deaconess Medical Center, Harvard Medical School, Boston, Massachusetts; 3 Department of Anesthesiology, Louisiana State University Health Sciences Center, Shreveport, Louisiana; 4 Valley Anesthesiology and Pain Consultants – Envision Physician Consultants, Phoenix, Arizona; 5 Department of Anesthesiology, University of Arizona College of Medicine – Phoenix, Phoenix, Arizona; 6 Department of Anesthesiology, Creighton University School of Medicine, Omaha, Nebraska; 7 Department of Anesthesiology, Perioperative and Pain Medicine, Brigham and Women's Hospital, Harvard Medical School, Boston, Massachusetts

**Keywords:** spinal cord stimulator, infection, interventional pain medicine

## Abstract

Spinal cord stimulation is an effective treatment modality for patients with numerous pain conditions. Although proven to be highly successful, device implantation does come with some inherent risks. One of the most challenging complications is perioperative infection. For most patients, a simple trial of oral antibiotics and in-office drainage of any superficial infectious material may be sufficient. Deeper infections with wound dehiscence necessitate device removal and intravenous antibiotic therapy. The question remains, if the device was previously providing pain relief for the patient, when is the appropriate time to reimplant the device after the infection has cleared? We describe the case of explantation of an infected device and successful reimplantation after 1 year.

Spinal cord stimulation (SCS) is an effective treatment modality for patients with chronic pain used by interventional pain medicine physicians: SCS has demonstrated clinical benefit in patients with complex regional pain syndrome, failed back surgery syndrome, critical limb ischemia, and refractory angina pectoris. 1 SCS therapy comes with the risk of potential complications including device failure, lead migration, loss of therapeutic paresthesia, and infection. 2 For most clinicians, the management of these complications involves removal of the device and immediate reimplantation, except in the case of an infection. Although only seen in ∼2.45% of SCS implants, infected devices are some of the most dreaded complications and add the risk of meningitis, epidural abscess, and vertebral osteomyelitis. 2 3 It is crucial for physicians to recognize potential infections in a timely manner to prevent these more serious adverse effects. After explantation and initiation of antibiotic coverage, the question remains; what is the appropriate time to wait to reimplant? We briefly describe the case of the removal of an infected SCS device and successful reimplantation after a year.

## Case Report

A 48-year-old female with past medical history of anemia, depression, and lumbar radiculopathy presented to the pain clinic for the evaluation of her chronic low back pain. She rated the pain as a constant 8/10 for the past few years. The pain radiated into her right buttock and right lateral thigh. Magnetic resonance imaging (MRI) of the lumbar spine revealed a small disc protrusion at the L5/S1 level indenting the ventral thecal canal without significant spinal stenosis. No nerve root impingement was seen either. She had tried many pain treatments including interlaminar and transforaminal epidural steroid injections, oral pain medications including gabapentin, Lyrica, tramadol, and Percocet, and physical therapy. She underwent successful percutaneous placement of a Boston Scientific spinal cord stimulator device to the T8/9 level using epidural leads.

Twelve days after implantation, she experienced poor wound healing. Upon inspection of her lower back, wound dehiscence was noted. An infectious workup was performed noting elevated white blood cell count (WBC), erythrocyte sedimentation rate (ESR), and C-reactive protein (CRP). She was initially started on cephalexin 500 mg orally three times a day for presumed cellulitis. The patient followed up a week later with erythema and purulent drainage of the incision site. Although she exhibited no systemic symptomatology, the decision was made to remove the device.

Intraoperative cultures were taken and she was started on vancomycin empirically. The wound was initially left open and she was admitted to the inpatient service. Cultures eventually came back positive for *Staphylococcus pseudintermedius* . An infectious disease consultation was obtained recommending a peripherally inserted central catheter (PICC) line and she underwent 6 weeks of outpatient intravenous Vancomycin therapy. The patient was apprehensive about reimplanting the device so she tried multimodal modal analgesics. Her pain was kept at bay for ∼8 months, but her symptomatology returned with greater force. Finally, after a year she presented to clinic again for spinal cord stimulator placement. The infection was deemed clear from her system and the device was reimplanted based on the immense success she previously had. To access the epidural space, we utilized the L1 level instead of the L2 level previously used to navigate around potential epidural adhesions. The device was placed smoothly, without difficulty. She regained paresthesia coverage with significant pain relief and to this date no signs of infection.

## Discussion

As with any implantable medical device, there is an inherent risk of bacterial colonization and resultant infection. The incidence of infection after SCS implantation is low and not associated with cancer, but rather increased surgical time. 4 Smoking, diabetes, malnutrition, poor hygiene, and pre-existing infection have all been associated with increased infectious risk. 5 Patients with diabetes are especially prone to surgical site infections and their hemoglobin A1c should be optimized preoperatively. Patients with smoking history are advised to stop smoking for at least 4 weeks before the operation. These patients may benefit from a nicotine patch in the interim. Certain infection sites like dental, skin, or urinary sites need to be clear of infection preoperatively. Postoperative occlusive dressings and oral antibiotics have been proven to decrease overall infection rates. 2

If an infection is suspected, a full history and physical exam must be performed to ensure no neurological symptomatology. MRI of the lumbar spine may be considered to rule out a potential epidural abscess. The most common infectious site is the generator pocket. 5 Superficial infections of the battery site might not warrant device removal, but deeper infections justify explantation, broad-spectrum antibiotic coverage, and a consultation from an infectious disease specialist. 6 *Staphylococcus* species and *Enterococcus* are commonly implicated in superficial surgical site infections with *Staphylococcus aureus* seen frequently in epidural abscess formations. 7

The surgical site must be cultured and sensitivities to antibiotics determined. If a deeper infection is suspected, like in our patient, a PICC line may be indicated for intravenous antibiotics over an extended period of time. To deem a patient cleared of the infection, the infection site may need to be recultured. Infectious biomarkers (WBC, ESR, CRP) should all be downtrending or normalized. The patient should look clinically well with no constitutional symptomatology. The surgical site should be clean and intact with no discharge, redness, or persistent dehiscence.

## Conclusion

There are no consensus guidelines for when a device can be reimplanted. 8 Each infection has a unique presentation based on the extent of inoculation. Deeper infections with neurological sequela often need extended intravenous antibiotic therapy for months and many clinicians may not recommend reimplantation. The most important indication for reimplantation is the resolution of infectious symptomatology for at least 90 days according to the Neurostimulation Appropriateness Consensus Committee (NACC). 8 If the decision is eventually made for reimplantation, a longer postoperative course of antibiotics may be advantageous. Further studies must be undertaken to determine consensus guidelines of antibiotic therapy duration and time to wait for reimplantation if deemed clinically appropriate.
